# Poly[diaqua­di-*μ*_4_-citrato-trizinc(II)]

**DOI:** 10.1107/S1600536808007642

**Published:** 2008-03-29

**Authors:** Jian Wu

**Affiliations:** a College of Chemistry and Ecological Engineering, Guangxi University for Nationalities, Nanning 530006, Guangxi, People’s Republic of China

## Abstract

The title compound, [Zn_3_(C_6_H_5_O_7_)_2_(H_2_O)_2_]_*n*_, is a polymer in which the repeating unit contains three zinc atoms, two hepta-dentate Hcit ligands (Hcit = citric acid trianion) and two coordinated water mol­ecules, only half of which are independent due to one of the metal atoms lying on a centre of symmetry. The two independent cations both exhibit an octa­hedral geometry, but the way in which they are coordinate are different; while the Zn atom in a general position is bound to three Hcit ligands and one water mol­ecule, the one at the centre of symmetry is coordinated by six O atoms from two symmetry-related Hcit ligands through the (protonated) hydroxyl and carboxyl­ate groups. The three carboxyl­ate groups coordinate to the Zn centres in three different ways, *viz*. chelating, bridging and a mixture of both, in an unusual coordination mode for citrate. The result is a two-dimensional structure parallel to (010), built up by a square-grid motif. Intermolecular O—H⋯O hydrogen bonds are present in the crystal structure

## Related literature

For related literature, see: Albrecht *et al.* (2000 ▶); Dybtsev *et al.* (2004 ▶); Lightfoot & Sueddden (1999 ▶); Ma *et al.* (2000 ▶); Xie *et al.* (2004 ▶, 2005 ▶); Yaghi & Li (1996 ▶); Yaghi & Rowsell (2006 ▶); Zhao *et al.* (2006 ▶); Zou *et al.* (2006 ▶).
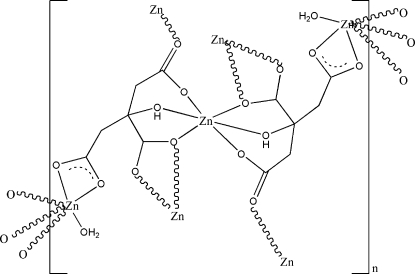


## Experimental

### 

#### Crystal data

[Zn_3_(C_6_H_5_O_7_)_2_(H_2_O)_2_]
*M* *_r_* = 610.34
Monoclinic, 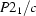

*a* = 6.1073 (5) Å
*b* = 15.3132 (12) Å
*c* = 9.7858 (8) Å
β = 102.79 (10)°
*V* = 892.48 (12) Å^3^
*Z* = 2
Mo *K*α radiation
μ = 4.09 mm^−1^
*T* = 298 (2) K
0.20 × 0.18 × 0.18 mm

#### Data collection

Bruker APEXII area-detector diffractometer
Absorption correction: multi-scan (*SADABS*; Sheldrick, 2004 ▶) *T*_min_ = 0.46, *T*_max_ = 0.48
4693 measured reflections
1749 independent reflections
1694 reflections with *I* > 2σ(*I*)
*R*_int_ = 0.026

#### Refinement

*R*[*F* ^2^ > 2σ(*F* ^2^)] = 0.048
*wR*(*F* ^2^) = 0.233
*S* = 1.36
1648 reflections
143 parameters
1 restraint
H-atom parameters constrained
Δρ_max_ = 1.18 e Å^−3^
Δρ_min_ = −1.06 e Å^−3^

### 

Data collection: *APEX2* (Bruker, 2004 ▶); cell refinement: *SAINT* (Bruker, 2004 ▶); data reduction: *SAINT*; program(s) used to solve structure: *SHELXS97* (Sheldrick, 2008 ▶); program(s) used to refine structure: *SHELXL97* (Sheldrick, 2008 ▶); molecular graphics: *SHELXTL* (Sheldrick, 2008 ▶) and *DIAMOND* (Brandenburg, 2004 ▶); software used to prepare material for publication: *SHELXTL*.

## Supplementary Material

Crystal structure: contains datablocks I, global. DOI: 10.1107/S1600536808007642/bg2172sup1.cif

Structure factors: contains datablocks I. DOI: 10.1107/S1600536808007642/bg2172Isup2.hkl

Additional supplementary materials:  crystallographic information; 3D view; checkCIF report

## Figures and Tables

**Table 1:** Selected bond lengths (Å)

Zn1—O7	2.270 (6)
Zn1—O4	2.285 (6)
Zn1—O3	2.319 (6)
Zn2—O5^i^	2.232 (7)
Zn2—O1*W*	2.244 (7)
Zn2—O6^ii^	2.316 (6)
Zn2—O2	2.338 (6)
Zn2—O1	2.371 (7)
Zn2—O7^ii^	2.485 (6)

Symmetry codes: (i) 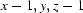; (ii) 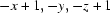.

**Table 2:** Hydrogen-bond geometry (Å, °)

*D*—H⋯*A*	*D*—H	H⋯*A*	*D*⋯*A*	*D*—H⋯*A*
O3—H3⋯O2^iii^	0.82	1.88	2.691 (8)	172
O1*W*—H1*WA*⋯O7^iv^	0.82	2.35	3.071 (10)	148
O1*W*—H1*WB*⋯O6^v^	0.82	2.00	2.811 (10)	171

Symmetry codes: (iii) 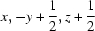; (iv) 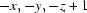; (v) 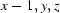.

**Fig. 1.**
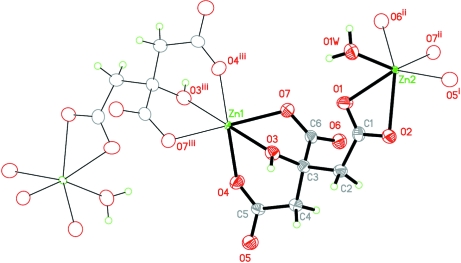
Molecular diagram of (I), showing 30% probability displacement ellipsoids. In bold, the asymmetric unit. Symmetry codes: (i) x - 1, y, z - 1; (ii) -x + 1, -y, -z + 1, (iii) -x + 1, -y, -z + 2.

**Fig. 2.**
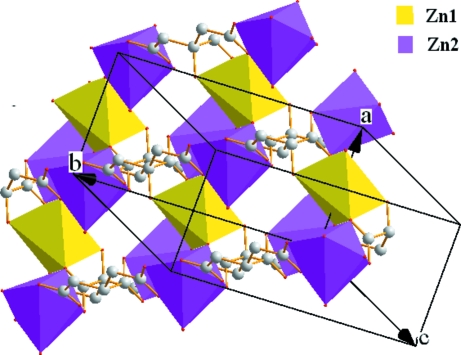
Two-dimensional network structure of complex (I). Colour codes: yellow, Zn1 polyhedra, pink, Zn2 polyhedra.

## supplementary crystallographic information

### Comment

The rational design and syntheses of novel coordination polymers have achieved considerable progress in the field of supramolecular chemistry and crystal engineering, owing to their potential applications as gas storage (Yaghi & Rowsell, 2006), sensor technology (Albrecht *et al.*, 2000), separation processes (Dybtsev *et al.*, 2004), ion exchange (Yaghi & Li, 1996), luminescence (Zhao *et al.*, 2006), magnetism (Ma *et al.*, 2000), and catalysis (Zou *et al.*, 2006), as well as due to their intriguing variety of architectures and topologies. Flexible di- and polycarboxylic acids are good candidates for the construction of novel metal-organic compounds as the carboxyl groups can form C—O—M—O four-membered rings with central metal ions, thereby improving the stability of transition metal-organic frameworks (MOFs). Furthermore, di- and polycarboxylic acids have two or more carboxyl groups that can be completely or partially deprotonated, which results in a rich variety of coordination modes and many interesting structures with higher dimensions. However, the hydroxyl polycarboxylates (HPCs), such as malate, citrate and tartrate, have been less studied as building blocks in the construction of metal-organic frameworks (Lightfoot & Sueddden,1999; Xie *et al.*, 2004, 2005). Hydroxypolycarboxylic acids can act not only as hydrogen-bond acceptors but also as hydrogen-bond donors, depending on the number of deprotonated carboxyl group. In all known citrate-bridging compounds, the oxygen atoms of the alkoxyl or hydroxyl groups participate the coordination, which allows the formation of five- and six-membered rings, stabilizing the solid networks. In this paper, we report the synthesis and crystal structure of the title complex,(I).

As shown in the Scheme and in Fig. 1, the compound is a polymer where the repeating unit contains three zinc atoms, two hepta-dentate Hcit ligand (Hcit =citric acid trianion) and two coordinated water molecules, only half of which are independent due to the Zn1 atom lying on centre of symmetry.Both cations present an octahedral geometry; Zn2 is bound to three Hcit ligands and one coordinated water molecule, while the centrosymmetric Zn1 is coordinated by six oxygens from two symmetry related Hcit groups through the (protonated) hydroxyl and carboxylate groups.

The mean Zn—O bond length is 2.26 (2)Å (Table.1).

The carboxylate groups bind to the Zn centres in three different ways. The first group (O1—O2) chelates Zn2, the second (O4—O5) adopts a Zn1—Zn2 bridging mode while the third (O6—O7) chelates Zn2 while serving as a Zn1—Zn2 bridge as well. As a result of this unusual coordination mode *via* its three carboxylates groups and the hydroxy group, each citrate molecule binds four different Zn centres, *viz*.: to Zn1, in a tridentate way, and to three symmetry related Zn2, in bidentate or bridging fashion (Fig. 1). The result is a two-dimensional structure parallel to (010), built up by a square grid motive with cavity dimension of about 6.10*5.10 Å (Fig.2).

### Experimental

ZnSO4(0.025 g, 0.013 mmol), citric acid(0.021 g, 0.016 mmol) and NaOH(0.048 mmol,0.12 mmol), were mixed in acetonitrile, and the mixture was heated for six hours under reflux. During the process stirring and influx were required. The resultant was then filtered to give a pure solution which was infiltrated by diethyl ether freely in a closed vessel. Four weeks later some single crystals of a suitable size for X-Ray diffraction analysis appeared.

### Refinement

The H atoms attached to carbon were placed in calculated positions [C–H = 0.97 Å] with *U*_iso_(H) = 1.2*U*_eq_(C). Those attached to oxygen were found in a difference map and adjusted so that O—H = 0.82 Å, with *U*_iso_(H) = 1.5*U*_eq_(C). All H atoms were allowed to ride onto their hosts.

### Crystal data

**Table:**

[Zn_3_(C_6_H_5_O_7_)_2_(H_2_O)_2_]	*F*_000_ = 608
*M**_r_* = 610.34	*D*_x_ = 2.271 Mg m^−^^3^
Monoclinic, *P*2_1_/*c*	Mo *K*α radiation λ = 0.71073 Å
Hall symbol: -P 2ybc	Cell parameters from 1749 reflections
*a* = 6.1073 (5) Å	θ = 2.5–26.0º
*b* = 15.3132 (12) Å	µ = 4.09 mm^−^^1^
*c* = 9.7858 (8) Å	*T* = 298 (2) K
β = 102.7910 (10)º	Block, colourless
*V* = 892.48 (12) Å^3^	0.20 × 0.18 × 0.18 mm
*Z* = 2	

### Data collection

**Table:**

Bruker APEXII area-detector diffractometer	1749 independent reflections
Radiation source: fine-focus sealed tube	1694 reflections with *I* > 2σ(*I*)
Monochromator: graphite	*R*_int_ = 0.026
*T* = 298(2) K	θ_max_ = 26.0º
φ and ω scan	θ_min_ = 2.5º
Absorption correction: multi-scan(SADABS; Sheldrick, 2004)	*h* = −4→7
*T*_min_ = 0.46, *T*_max_ = 0.48	*k* = −18→18
4693 measured reflections	*l* = −12→11

### Refinement

**Table:**

Refinement on *F*^2^	Secondary atom site location: difference Fourier map
Least-squares matrix: full	Hydrogen site location: inferred from neighbouring sites
*R*[*F*^2^ > 2σ(*F*^2^)] = 0.048	H-atom parameters constrained
*wR*(*F*^2^) = 0.233	*w* = 1/[σ^2^(*F*_o_^2^) + (0.160*P*)^2^ + 1.0412*P*] where *P* = (*F*_o_^2^ + 2*F*_c_^2^)/3
*S* = 1.36	(Δ/σ)_max_ < 0.001
1648 reflections	Δρ_max_ = 1.18 e Å^−^^3^
143 parameters	Δρ_min_ = −1.06 e Å^−^^3^
1 restraint	Extinction correction: SHELXL, Fc^*^=kFc[1+0.001xFc^2^λ^3^/sin(2θ)]^-1/4^
Primary atom site location: structure-invariant direct methods	Extinction coefficient: 0.021 (6)

### Special details

**Table:**

Geometry. All e.s.d.'s (except the e.s.d. in the dihedral angle between two l.s. planes) are estimated using the full covariance matrix. The cell e.s.d.'s are taken into account individually in the estimation of e.s.d.'s in distances, angles and torsion angles; correlations between e.s.d.'s in cell parameters are only used when they are defined by crystal symmetry. An approximate (isotropic) treatment of cell e.s.d.'s is used for estimating e.s.d.'s involving l.s. planes.
Refinement. Refinement of *F*^2^ against ALL reflections. The weighted *R*-factor *wR* and goodness of fit *S* are based on *F*^2^, conventional *R*-factors *R* are based on *F*, with *F* set to zero for negative *F*^2^. The threshold expression of *F*^2^ > σ(*F*^2^) is used only for calculating *R*-factors(gt) *etc*. and is not relevant to the choice of reflections for refinement. *R*-factors based on *F*^2^ are statistically about twice as large as those based on *F*, and *R*- factors based on ALL data will be even larger.Some reflections data are omiited may attributted to their bad reflections.

### Fractional atomic coordinates and isotropic or equivalent isotropic displacement parameters (Å^2^)

**Table:**

	*x*	*y*	*z*	*U*_iso_*/*U*_eq_	
Zn1	0.5000	0.0000	1.0000	0.0150 (5)	
Zn2	0.11561 (9)	0.09674 (4)	0.31681 (6)	0.0129 (5)	
O1W	−0.1974 (13)	0.0915 (4)	0.4010 (8)	0.0543 (17)	
H1WA	−0.2908	0.0542	0.3686	0.081*	
H1WB	−0.1748	0.0841	0.4860	0.081*	
O1	0.3216 (12)	0.0872 (4)	0.5521 (7)	0.0506 (17)	
O2	0.3258 (10)	0.2105 (4)	0.4410 (7)	0.0473 (14)	
O3	0.4296 (10)	0.1275 (4)	0.8697 (6)	0.0427 (13)	
H3	0.3866	0.1764	0.8859	0.064*	
O4	0.8310 (11)	0.0722 (5)	1.0802 (7)	0.0504 (16)	
O5	1.0418 (12)	0.1871 (5)	1.1333 (7)	0.0566 (19)	
O6	0.8360 (11)	0.0532 (4)	0.6858 (6)	0.0471 (15)	
O7	0.6177 (11)	−0.0177 (4)	0.7974 (7)	0.0464 (15)	
C1	0.3868 (13)	0.1633 (5)	0.5492 (8)	0.0383 (17)	
C2	0.5471 (15)	0.2027 (5)	0.6762 (9)	0.0408 (19)	
H2A	0.4776	0.2546	0.7046	0.049*	
H2B	0.6825	0.2210	0.6478	0.049*	
C3	0.6146 (12)	0.1423 (6)	0.8041 (8)	0.0349 (17)	
C4	0.8172 (15)	0.1853 (6)	0.9036 (9)	0.0419 (19)	
H4A	0.9423	0.1854	0.8574	0.050*	
H4B	0.7793	0.2458	0.9168	0.050*	
C5	0.8961 (12)	0.1437 (5)	1.0478 (8)	0.0356 (16)	
C6	0.6918 (13)	0.0533 (5)	0.7569 (8)	0.0360 (17)	

### Atomic displacement parameters (Å^2^)

**Table:**

	*U*^11^	*U*^22^	*U*^33^	*U*^12^	*U*^13^	*U*^23^
Zn1	0.0192 (7)	0.0147 (7)	0.0113 (7)	−0.0059 (3)	0.0041 (4)	0.0027 (3)
Zn2	0.0151 (6)	0.0123 (6)	0.0095 (6)	0.0008 (2)	−0.0014 (4)	0.0000 (2)
O1W	0.066 (4)	0.056 (4)	0.044 (4)	−0.001 (3)	0.018 (3)	−0.004 (3)
O1	0.061 (4)	0.038 (3)	0.044 (4)	−0.009 (3)	−0.008 (3)	0.005 (3)
O2	0.054 (4)	0.047 (3)	0.036 (3)	−0.007 (3)	0.000 (3)	0.002 (2)
O3	0.049 (3)	0.037 (3)	0.042 (3)	0.007 (3)	0.009 (3)	−0.003 (3)
O4	0.053 (4)	0.051 (4)	0.043 (4)	−0.015 (3)	−0.001 (3)	0.011 (3)
O5	0.060 (4)	0.055 (4)	0.046 (4)	−0.012 (3)	−0.007 (3)	0.008 (3)
O6	0.048 (3)	0.048 (4)	0.047 (3)	0.001 (3)	0.016 (3)	−0.002 (3)
O7	0.059 (4)	0.039 (3)	0.042 (3)	−0.005 (3)	0.012 (3)	0.003 (3)
C1	0.039 (4)	0.040 (4)	0.036 (4)	0.006 (3)	0.008 (3)	0.006 (3)
C2	0.047 (5)	0.033 (4)	0.040 (5)	−0.003 (3)	0.005 (4)	−0.001 (3)
C3	0.035 (4)	0.037 (4)	0.031 (4)	0.002 (3)	0.003 (3)	0.001 (3)
C4	0.045 (5)	0.041 (4)	0.038 (5)	−0.006 (3)	0.006 (4)	−0.003 (3)
C5	0.035 (4)	0.034 (4)	0.035 (4)	−0.002 (3)	0.002 (3)	−0.004 (3)
C6	0.036 (4)	0.041 (4)	0.029 (4)	−0.001 (3)	0.003 (3)	0.001 (3)

### Geometric parameters (Å, °)

**Table:**

Zn1—O7	2.270 (6)	O3—H3	0.82
Zn1—O7^i^	2.270 (6)	O4—C5	1.231 (10)
Zn1—O4^i^	2.285 (6)	O5—C5	1.266 (10)
Zn1—O4	2.285 (6)	O5—Zn2^iv^	2.232 (7)
Zn1—O3^i^	2.319 (6)	O6—C6	1.237 (9)
Zn1—O3	2.319 (6)	O6—Zn2^iii^	2.316 (6)
Zn2—O5^ii^	2.232 (7)	O7—C6	1.273 (10)
Zn2—O1W	2.244 (7)	O7—Zn2^iii^	2.485 (6)
Zn2—O6^iii^	2.316 (6)	C1—C2	1.526 (11)
Zn2—O2	2.338 (6)	C2—C3	1.538 (11)
Zn2—O1	2.371 (7)	C2—H2A	0.9700
Zn2—O7^iii^	2.485 (6)	C2—H2B	0.9700
O1W—H1WA	0.8200	C3—C4	1.543 (11)
O1W—H1WB	0.8200	C3—C6	1.546 (11)
O1—C1	1.233 (10)	C4—C5	1.526 (11)
O2—C1	1.268 (10)	C4—H4A	0.9700
O3—C3	1.435 (9)	C4—H4B	0.9700
			
O7—Zn1—O7^i^	180.000 (1)	C3—O3—H3	105.1
O7—Zn1—O4^i^	94.0 (2)	Zn1—O3—H3	133.7
O7^i^—Zn1—O4^i^	86.0 (2)	C5—O4—Zn1	130.9 (5)
O7—Zn1—O4	86.0 (2)	C5—O5—Zn2^iv^	101.2 (5)
O7^i^—Zn1—O4	94.0 (2)	C6—O6—Zn2^iii^	96.6 (5)
O4^i^—Zn1—O4	180.0	C6—O7—Zn1	111.9 (5)
O7—Zn1—O3^i^	108.9 (2)	C6—O7—Zn2^iii^	87.8 (5)
O7^i^—Zn1—O3^i^	71.1 (2)	Zn1—O7—Zn2^iii^	144.8 (3)
O4^i^—Zn1—O3^i^	79.9 (2)	O1—C1—O2	121.4 (8)
O4—Zn1—O3^i^	100.1 (2)	O1—C1—C2	120.5 (7)
O7—Zn1—O3	71.1 (2)	O2—C1—C2	118.1 (7)
O7^i^—Zn1—O3	108.9 (2)	C1—C2—C3	115.6 (7)
O4^i^—Zn1—O3	100.1 (2)	C1—C2—H2A	108.4
O4—Zn1—O3	79.9 (2)	C3—C2—H2A	108.4
O3^i^—Zn1—O3	180.000 (1)	C1—C2—H2B	108.4
O5^ii^—Zn2—O1W	106.3 (3)	C3—C2—H2B	108.4
O5^ii^—Zn2—O6^iii^	127.5 (3)	H2A—C2—H2B	107.4
O1W—Zn2—O6^iii^	95.1 (2)	O3—C3—C2	111.3 (6)
O5^ii^—Zn2—O2	86.9 (2)	O3—C3—C4	112.7 (6)
O1W—Zn2—O2	104.5 (2)	C2—C3—C4	106.8 (7)
O6^iii^—Zn2—O2	133.4 (2)	O3—C3—C6	108.5 (6)
O5^ii^—Zn2—O1	142.1 (2)	C2—C3—C6	109.4 (6)
O1W—Zn2—O1	87.3 (3)	C4—C3—C6	108.1 (6)
O6^iii^—Zn2—O1	84.7 (2)	C5—C4—C3	116.7 (7)
O2—Zn2—O1	55.2 (2)	C5—C4—H4A	108.1
O5^ii^—Zn2—O7^iii^	88.6 (3)	C3—C4—H4A	108.1
O1W—Zn2—O7^iii^	147.6 (2)	C5—C4—H4B	108.1
O6^iii^—Zn2—O7^iii^	54.1 (2)	C3—C4—H4B	108.1
O2—Zn2—O7^iii^	104.9 (2)	H4A—C4—H4B	107.3
O1—Zn2—O7^iii^	98.5 (2)	O4—C5—O5	121.1 (7)
Zn2—O1W—H1WA	117.3	O4—C5—C4	123.8 (7)
Zn2—O1W—H1WB	114.3	O5—C5—C4	115.1 (7)
H1WA—O1W—H1WB	104.0	O6—C6—O7	121.4 (8)
C1—O1—Zn2	91.4 (5)	O6—C6—C3	118.1 (7)
C1—O2—Zn2	92.0 (5)	O7—C6—C3	120.4 (7)
C3—O3—Zn1	108.1 (4)		

Symmetry codes: (i) −*x*+1, −*y*, −*z*+2; (ii) *x*−1, *y*, *z*−1; (iii) −*x*+1, −*y*, −*z*+1; (iv) *x*+1, *y*, *z*+1.

### Hydrogen-bond geometry (Å, °)

**Table:**

*D*—H···*A*	*D*—H	H···*A*	*D*···*A*	*D*—H···*A*
O3—H3···O2^v^	0.82	1.88	2.691 (8)	172
O1W—H1WA···O7^vi^	0.82	2.35	3.071 (10)	148
O1W—H1WB···O6^vii^	0.82	2.00	2.811 (10)	171

Symmetry codes: (v) *x*, −*y*+1/2, *z*+1/2; (vi) −*x*, −*y*, −*z*+1; (vii) *x*−1, *y*, *z*.
